# Supplementary material for: Complexome profiling of the Chlamydomonas psb28 mutant reveals TEF5 as an early PSII assembly factor
Source: Plant Cell. 2025 Mar 17;37(6):koaf055. doi: 10.1093/plcell/koaf055 (PMC12164590; doi:10.1093/plcell/koaf055)
Supplement: koaf055_Supplementary_Data [file koaf055_supplementary_data.zip › psb28_interactive.zip!/plots/intensity/qualityPlots/peptides/_Cre06.g290350.t1.2.html]

### Cre06.g290350.t1.2

Trivial name:   
SeqCoverage [median]: 16.80  
Mass: 19.43 kDa  
  
 

| Sequence | MissedCleavage | Mass [Da] | wtIntensity | muIntensity | Score |
| --- | --- | --- | --- | --- | --- |
| KDETAATAAAATLK | 1 | 1360.72 | 0.00 | 454.27 | 98.59 |
| LAAPGAVLPK | 0 | 935.58 | 521.31 | 1527.48 | 75.24 |
| VLVESIR | 0 | 814.49 | 0.00 | 594.36 | 76.03 |
|  |  |  | 521.3 | 2576.1 |  |
